# Comorbidities of epilepsy in low and middle-income countries: systematic review and meta-analysis

**DOI:** 10.1038/s41598-020-65768-6

**Published:** 2020-06-02

**Authors:** Aline Muhigwa, Pierre-Marie Preux, Daniel Gérard, Benoit Marin, Farid Boumediène, Charles Ntamwira, Chung-Huang Tsai

**Affiliations:** 1 INSERM, IRD associated unit, U1094, Neuroépidémiologie Tropicale, Institut d’Epidémiologie et de Neurologie Tropicale, CHU Limoges, GEIST, 87000 Limoges, France; 2 Faculté de médecine, Université officielle de Bukavu/1, Avenue Kasongo, Commune d’Ibanda, B.P. 570 Bukavu, Democratic Republic of the Congo; 3 Department of family medicine, Chung-Kang Branch, Cheng Ching hospital, Taiwan No.966.sec. 4, Taiwan Blvd. Xitun Dist., Taichung, Taiwan, ROC

**Keywords:** Medical research, Risk factors

## Abstract

Epilepsy is a major public health concern in low and middle-income countries (LMICs) and comorbidities aggravate the burden associated with the disease. The epidemiology of these comorbidities has not been well described, although, identifying the main comorbidities of epilepsy, and their relative importance, is crucial for improving the quality of care. Comorbidities were defined as disorders coexisting with or preceding epilepsy, or else compounded or directly attributed to epilepsy or to its treatment. A meta-analysis of the proportion of main comorbidities by subcontinent as well as overall was also conducted. Out of the 2,300 papers identified, 109 from 39 countries were included in this systematic review. Four groups of comorbidities were identified: parasitic and infectious diseases (44% of comorbid conditions), somatic comorbidities (37%), psychosocial (11%), as well as psychiatric comorbidities (8%). Heterogeneity was statistically significant for most variables then random effect models were used. The most frequently studied comorbidities were: neurocysticercosis (comorbid proportion: 23%, 95% CI: 18–29), head trauma (comorbid proportion: 9%, 95% CI: 5–15) malnutrition (comorbid proportion: 16%, 95% CI: 28–40), stroke (comorbid proportion: 1.3%, 95% CI: 0.2–7.0), and discrimination for education (comorbid proportion: 34%, 95% CI: 28–40). Many comorbidities of epilepsy were identified in LMICs, most of them being infectious.

## Introduction

Epilepsy is characterized by the recurrence of seizures with neurological, cognitive, psychological and social impacts. A seizure is the transient presence of signs and/or symptoms due to a synchronous abnormal or excessive neural activity in the brain^1^.

In developed countries, the overall prevalence of epilepsy ranges from 5‰ to 8‰^2–4^ whereas in low- and middle-income countries, except in Asia, the proportion is 2–3 times higher^5,6^. As a consequence, among the 70 million people living with epilepsy in the world, 85% are found in countries with low income^7^.

Epilepsy is a major public health issue due to its medical, social, cultural and economic consequences^8,9^. It carries a significant burden not only due to the seizures themselves, but also to the comorbidities, the disabilities, and the stigma associated with the disease^10^.

In developed countries, over the last decade, researchers have established reliable and valid measures in PWE^11^. They explored comorbidities and grouped them into two large entities: psychiatric and somatic. In Texas, USA, an estimated 41% of PWE presented psychiatric and somatic disorders when compared to 15–20% in the general population; In England the proportion of PWE with comorbidities was 32%^12^. Tellez-Zenteno *et al*.^13^, using the Composit International Diagnostic Interview (CIDI) in a descriptive epidemiological survey in communities across Canada, diagnosed psychiatric disorders in 35% of PWE, compared to 20% in people without epilepsy. A cohort study in 713 adults with epilepsy in Sweden reported a comorbidity proportion of 5.9% for psychiatric disorders and 50% for somatic disorders^14^.

A link between epilepsy and certain comorbidities such as depression, attention deficit with hyperactivity disorder, anxiety, migraine, sleep disorders and malnutrition may exist through a common etiology or common genetic or environmental factors, as well as side effects of anti-epileptics^15^. Delay in starting epilepsy treatment is present in about 70% of cases where there is an occurrence of comorbidities^16^.

The epidemiology of the comorbidities of epilepsy in LMICs, as well as the link between these disorders and epilepsy have not been well described. There is no overall information on comorbidities affecting PWE in LMICs in the existing literature, and the data on the proportion of various comorbidities of epilepsy are fragmented. However, a good knowledge of epilepsy comorbidities is crucial for an accurate diagnosis and for delivering appropriate and comprehensive care, especially in LMICs where medical resources are scarce. Thus, we conducted a systematic review of studies on comorbidities of epilepsy in LMICs.

## Methods

We referred to the recommendations of the PRISMA statement^17^ for drafting systematic reviews and meta-analysis in epidemiology.

### Definitions

The co-occurrence of various conditions is amplified in the context of chronic diseases and the understanding of how these relate to each other is essential. Feinstein defined a comorbidity as “any separate additional clinical entity that exists (…) in the clinical course of a patient who has the index of a disease”. Comorbidity is a broad concept, including complications, causes, signs, or symptoms^18^. It is currently set by the experts as a disorder coexisting with epilepsy and which may precede, be compounded or be directly attributable to epilepsy or its treatment^19,20^. Comorbidities were split in 4 groups: psychiatric comorbidities, psychosocial comorbidities (education discrimination, marriage discrimination…), somatic comorbidities, and infectious - in particular parasitic- comorbidities.

### Search strategy

**Figure 1:**
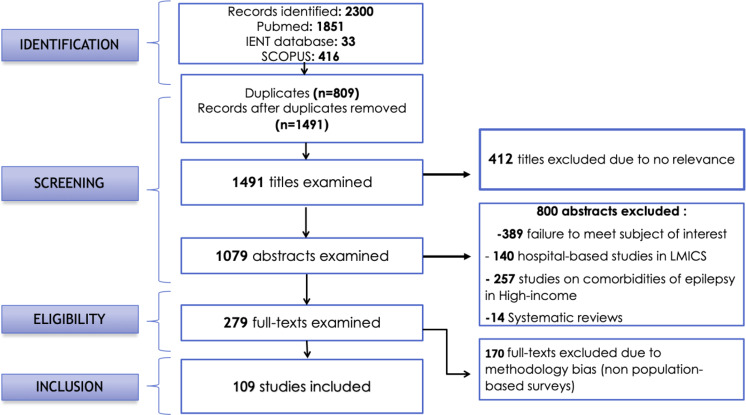
Flow-chart illustrating the selection procedure.

We conducted a systematic review in 3 databases: Pubmed/Medline, IENT (Institute of Epidemiology and Tropical Neurology – https://www.unilim.fr/ient) and Scopus. English keywords were used. Regarding Pubmed/Medline research the word “epilepsy” was combined with each of 62 comorbidities and was evaluated among the countries categorized as LMICs by the World Bank in 2015. At a second level the word “epilepsy” was combined with each of the 16 chapters of the international classification of Diseases (ICD-9) and each of the LMICs. We adapted this research requirements to Scopus and IENT databases. The steps followed for the selection of the studies are illustrated in the flow chart (Fig. 1). Meta-analyses and systematic reviews were used as additional sources for identifying relevant studies.

### Inclusion criteria

To be included in our study, an article had to be conducted in general population in a LMIC setting, according to a cohort, cross-sectional or case-control methodology, and to include original data on epilepsy comorbidities.

### Exclusion criteria

Any study based on hospital data was excluded, as well as meta-analyses and systematic reviews.

### Data extraction

In each of the papers the following data were collected: the authors, the study year, the publication year, the geographic location, the study type, the methods, the population (rural, urban, suburban), the sample size and the subgroups by sex, age classes, type of epilepsy and type of comorbidities.

### Data analysis

A comorbidity included in the systematic review was considered for meta-analysis, if its link to epilepsy was physio-pathologically proven and if more than 3 studies were found on the subject. A meta-analysis of comorbidity proportions was performed using Stata software (Version 12). For each comorbidity, a ‘forest plot’ chart was produced. Then, a meta-analysis, global and stratified by sub-continents, was conducted. The pooled proportions were calculated. Data items were weighted in the computations. The weights were based on a precision estimator for each study, i.e. the opposite of the standard error of the proportion. We also calculated the I² index, which reflects the percentage of the total variation in all of the studies due to heterogeneity rather than random. Because heterogeneity was statistically significant, random effects models were used. To stabilize the variances of the proportions used in the calculations, the proportions used underwent a Logit transformation^21^. GPS (Global Positioning System) coordinates of the various study areas were searched http://www.gpsfrance.net/adresse-vers-coordonnees-gps enabling us to map the study sites.

### Ethics

This paper does not concern directly PWE but makes use of publications concerning comorbidities of epilepsy. Informed consent is not applicable. No approval of an ethics committee is applicable is this case of the figures.

## Results

**Figure 2:**
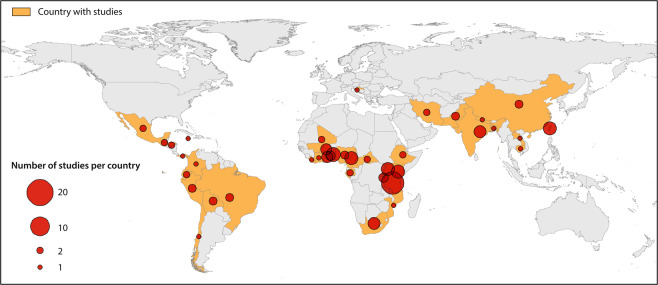
Distribution of studies on comorbidities of epilepsy in low- and middle-income countries.

A total of 2300 publications were identified and 109 from 39 countries were included in our study. The selection process is illustrated in the flowchart (Fig. 1) and Fig. 2 shows the worldwide distribution of the included studies.

**Figure 3:**
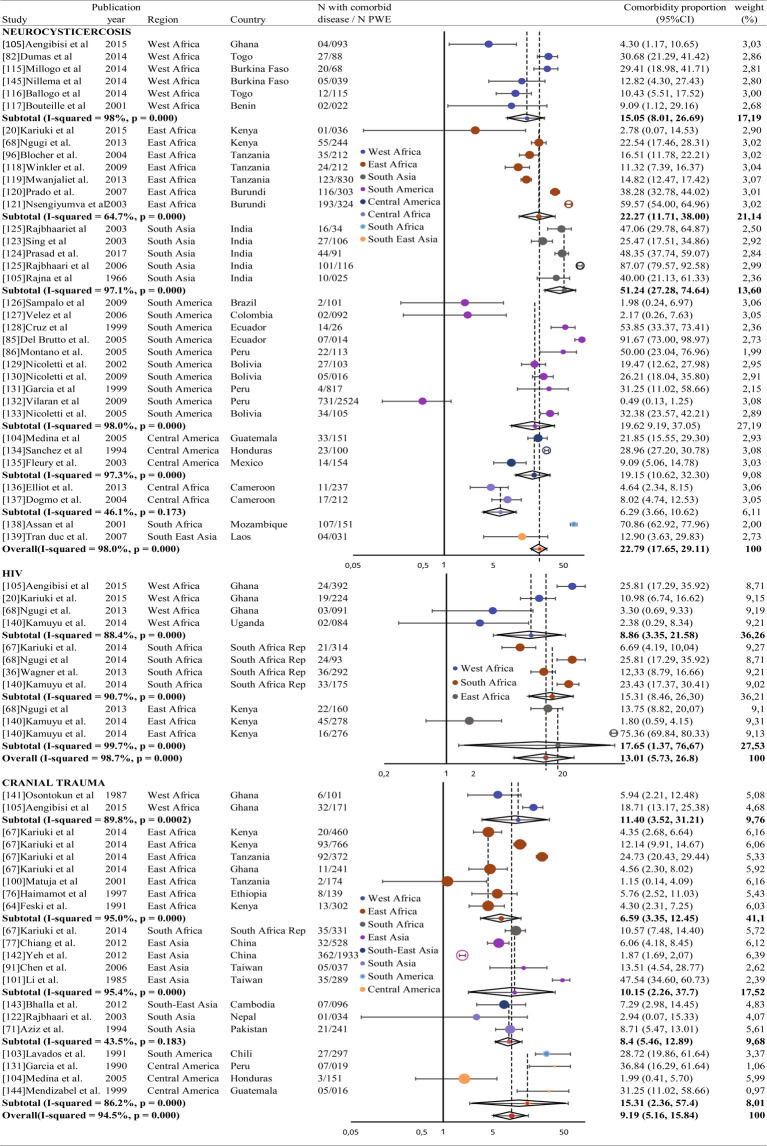
Forest plots of comorbidities in epilepsy in low- and middle-income countries (1/2).

**Figure 4:**
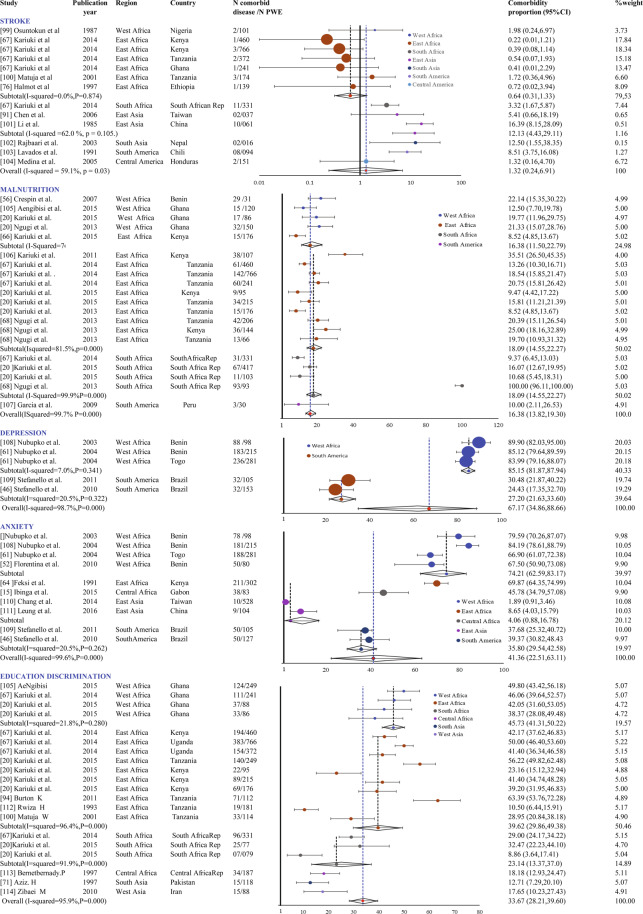
Forest plots of comorbidities in epilepsy in low- and middle-income countries (2/2).

Overall, 44% of studies concerned parasitic and infectious comorbidities, 37% somatic comorbidities, 11% psychosocial comorbidities and 8% psychiatric comorbidities. Criteria for meta-analysis were met for neurocysticercosis (assessed in 35 studies), cranial trauma (18 studies), stroke (10 studies), malnutrition (9 studies), depression (4 studies) and anxiety (9 studies), HIV (5 studies) and education discrimination (9 studies). Results per comorbidity, overall and stratified by continent, are presented in Figs. 3 and 4. A significant heterogeneity between studies was found for malnutrition (I^2^ test: 99.7%, Q test: p < 0.0001), depression (I^2^ test = 98.7%, Q test: p < 0.0001) and anxiety (I2 = 99.6%, Q test p < 0.0001). The estimates were based on random effects models. It is important to note that, regarding HIV, the overall comorbidity proportion is only valid for sub-Saharan Africa. Results about comorbidities included in the review, but not in the meta-analysis, are described below:

### Onchocerciasis

Thirteen studies evaluating comorbidity between epilepsy and onchocerciasis were identified. In East Africa the proportions varied between 81.8% and 98.6%^22–27^. In Central Africa only two studies were conducted with comorbidity proportion ranging from 22.4% to 36.9%. In West Africa the comorbidity proportions varied between 0 and 22.4%^28–33^.

### Meningitis

Only 3 studies have been carried out to identify the link between meningitis and epilepsy. The comorbidity proportions varied between 2.2% and 3.9%.

### Perinatal events

Twenty-six studies were identified (Supplementary Table 2). Perinatal trauma seems to be the most studied as 17 studies focused on the subject.

### High blood pressure

Several studies revealed high blood pressure in PWE varying from 5.4% to 41.9%^34–39^.

### Diabetes

Only two studies were identified with comorbidity proportions ranging from 5.3%^40^ to 26.4%^41^.

### Employment discrimination

It was assessed in 7 studies, with comorbidity proportions between 27.4%^42^ and 78.0%^22^ (Supplementary Table 1)

### Marriage discrimination

Eight studies were retrieved; the comorbidity proportions were between 46.6%^43^ and 88.9%^44^ (Supplementary Table 1).

### Other diseases

Migraine,1 study, comorbidity proportion: 2.7%^45^; brain tumor: 11 studies, comorbidity proportion between 0.6% and 9.0%; Fractures: 1 study, comorbidity proportion: 10.4%^45^; Burns: 4 studies, comorbidity proportions between 0.6% and 9.0%^22,46–48^; mental retardation: 4 studies, comorbidity proportion between 7.9% and 11.4%^45,47,49^; cognitive disorders: 4 studies, comorbidity proportion between 30.6% and 69.3%^22,48,50,51^ and sickle cell anemia: 1 study, comorbidity proportion:1.7%^8^.

## Discussion

### Psychiatric comorbidities

It is commonly accepted that patients with epilepsy are at a greater risk of developing psychiatric disorders^38^ but there are very few studies conducted in general population evaluating the proportion of psychiatric comorbidities in PWE in LMICs. The wide variation of comorbid proportions between studies may be due to methodological differences between the studies, and to the variability in the sample sizes, as well as the difference between the diagnostic instruments used.

#### Depression

The results of this meta-analysis are in line with (and even higher than) the data reported in developed countries where the common rate of depression in PWE was high. This could be explained by the limited availability of antiepileptic drugs in LMICs. Among the risk factors for depression in PWE, the frequency of seizures and the lack of treatments are prevalent^39^. The link between frequency of seizures and mental illness was also found in 696 patients with epilepsy in a study by Jacoby *et al*.^52^. In Europe the overall proportion of depression in PWE is estimated to be between 20% to 30%. Thus, depression would be the most frequent psychiatric comorbidity, affecting 20% to 50% of PWE in interictal period^53^. Some authors suggest a two-way relationship between depression and epilepsy^10,54^, likely due to neuroaminergic malfunctions common to depression and epilepsy^55^. Furthermore, depression is more prevalent in patients with uncontrolled epilepsy.

#### Anxiety

There is insufficient information on the proportion of anxiety comorbid to epilepsy in LMICs. Pooled results from our study show a high proportion of anxiety compared to figures reported in developed countries. Studies in developed countries have paid more attention to depression, although anxiety could be more frequent^56–58^ and it is known that both disorders often co-exist^42^. In some circles of specialized care, the comorbid proportion of anxiety disorders may exceed 50%^59–61^.

### Psychosocial factors

Even if it is not a disease, education discrimination can also be considered as a comorbidity because it is directly attributable to epilepsy. Education discrimination can be also explained by cognitive problems and behavioral issues which are frequent in epilepsy. They can have multiple causes, the most important being brain lesions, seizures, epileptic dysfunction, and treatment. Various social and cultural aspects in LMICS are harmful for PWE and are a source of stigma. It can be due to psychiatric disorders such as depression, anxiety or even suicide because the individual may feel useless, which would affect their self-esteem. This discrimination seems more accentuated in Africa than in Asia, and that could be explained by strong sociocultural restrictions hindering the education of PWE as they are considered as burdens to the community^62^. Because of the social weight, many children live hidden and cannot attend school, and in some cases, parents are forced to look for a school away from home, or even of changing schools to hide their child’s disease. Ultimately, children end up dropping out of school^50^. These psychosocial problems are critical obstacles in the management of epilepsy because of the public’s negative attitude towards PWE^63^.

### Somatic comorbidities

#### Cranial trauma

The comorbid proportion of traumatic brain injury in LMICS could be explained by the fact that road accidents are the main causes of injury, especially in Africa. They can also be linked to work related accidents, war wounds or uncontrolled falls during seizures in PWE^46^ or falling while walking on slippery muddy paths. The risk of developing post-traumatic epilepsy depends on the degree and the severity of the trauma as well as the resulting complications. In a study conducted in 2000 by Farnarier in Mali, post-traumatic epilepsy represented 7% of the total patients with epilepsy^2^.

According to Hauser, head trauma was one of the most important risk factors for epilepsy in a general population study that he conducted in Rochester, USA. Injury was identified as the cause of epilepsy in 6% of the population^64,65^. Assumptions have been made concerning the mechanisms of the occurrence of epilepsy after head trauma, including the deposit of iron associated with extravasation of blood, the increase in excitotoxicity due to the accumulation of glutamate, and diffuse axonal injury edema or ischemia^12^, to name a few.

#### Stroke

Epilepsy can be an early or late complication of stroke, which is one of the most common causes of epilepsy especially in the elderly. The overall pooled comorbidity proportion from our meta-analysis seems to be underestimated when compared to comorbidity proportions found in developed countries where investigation means are more sophisticated.

#### Malnutrition

Although malnutrition is not considered a direct cause of epilepsy, it seems to favor the onset of epilepsy or convulsions through various nutritional deficiencies. In LMICs, the relationship between epilepsy and malnutrition has been long suspected as a potential cause^66^.

It has been reported by some authors that the link between epilepsy and malnutrition in developing countries is difficult to establish because few studies have explored this potential relationship, with different methods conducting to inhomogeneous conclusions^66^.There are several hypotheses about the possible mechanisms such as the frequency of the number of seizures which could favorize malnutrition, or the decrease of immunity, due to malnutrition^66–68^, that could be involved in the occurrence of epilepsy. Biochemical changes due to malnutrition, such as electrolyte abnormalities and hypoglycemia could impact on the number of occurrences^69^.

On the other hand, due to the attitudes towards epilepsy in sub-Saharan Africa, epilepsy can also contribute to malnutrition as people with epilepsy are often victims of food taboos that could result in malnutrition in people with epilepsy. In a study conducted by Nubukpo^70^, for instance, 64% of people with epilepsy in Benin and 44% in Togo were victims of food taboos.

#### Perinatal events

Some studies have shown that the proportion of perinatal etiologies varies from 2%to 65% of the cases of epilepsy in sub-Saharan Africa^71–73^. Our literature search identified perinatal trauma as one of the most common causes of epilepsy in LMICs. This is consistent with what has been described by other systematic reviews including one conducted by Ba Diop *et al*.^4^. The legacy of birth injuries, often caused by a difficult birth, could lead to epilepsy within various time lapses^4^. It is difficult to prematurely attach the cause of seizure to a pre-, peri- or post-natal event because this link is often only based on the examination of the subject, or possibly on the interview of relatives, and thus is subject to biases of memorization^8^. Tradition or even the great distance from some villages to health centers sometimes force women to undergo childbirth at home without skilled assistance^74^.

#### High blood pressure

The comorbidity proportion found in different studies appears to be higher when compared to the general population. A meta-analysis of studies on the proportion of hypertension in the general population in Nigeria reported a rate between 12.4% and 34.8% in people without epilepsy^75^.

The range found in our study is substantially higher than that described in a larger study in the general population in the United Kingdom whose comorbidity proportion varied between 1.45% and1.95%^10^. This might be explained by the lifestyle of patients with epilepsy in LMICs in terms of food hygiene and permanent stress, which can predispose the individual to hypertension leading to seizures.

The relationship between hypertension and epilepsy is an indirect causal relationship (hypertension is a major risk factor for stroke), although some authors posit that high blood pressure might have an independent effect on epilepsy^75^.

#### Diabetes

The association of diabetes, especially type 1, and epilepsy has already been mentioned. However, the pathophysiology of this association has not yet been well elucidated^76^. Type 1 diabetes called diabetes mellitus or MODY (Maturity-Onset Diabetes of the Young) is observed in youth population with a proportion of 0.95% in the under 20 years age group^77^. Several mechanisms may play a role such as autoimmunity (antibodies to Glutamic Acid Decarboxylase) and genetics^76–78^. In our systematic review the proportions in LMICs seem obviously higher than that described in developed countries. However, it is not possible to draw a conclusion from only two isolated studies.

Brain tumors, paradoxically, have a very low comorbidity proportion in LMICs compared to developed countries, probably due to the lack of means of imaging exploration. Because of this obvious underestimation we did not include this comorbid condition although it met the criteria for meta-analysis.

Other somatic comorbidities such as burns, mental delays, cognitive disorders^18,45,79^ and sickle cell disease^8^ were also identified as being common in PWE in LMICs. Very few studies on the comorbid relation of these diseases with epilepsy were retrieved. This has not allowed us to perform a meta-analysis.

### Infectious and parasitic comorbidities

In LMICS, such as those in sub-Saharan Africa or Latin America, infections seem to explain the high proportion of epilepsy^3^. The Commission on tropical diseases of the International League against epilepsy has listed several diseases as being the causes of epilepsy, including malaria, tuberculosis, schistosomiasis, HIV/AIDS and neurocysticercosis. They seem to be the most frequent causes of epilepsies in a tropical environment^80^.

#### Neurocysticercosis

Cysticercosis is the most common neurological infection and is described in the literature as being a major cause of epilepsy in the tropics, particularly in Asia and Latin America^81,82^. It is associated with 30–50% of epilepsies in endemic areas like Peru where almost half the population lives in conditions where the transmission of *Taenia solium* is endemic^83,84^.

#### Onchocerciasis

Studies have confirmed an association between the proportion of filariasis such as the Loa loa, or the bancroftian infection, and the occurrence of seizures, but their causal effects or association with epilepsy are controversial^85,86^. The association between river blindness and epilepsy is still a matter of debate to date because there is a discrepancy between the results of different studies on the subject^71^.

#### HIV infection

Very few studies were conducted in the general population in LMICs on the comorbidity between epilepsy and HIV. Studies on this association are mostly outdated and from hospital series which are sources of substantial bias. We included HIV in the meta-analysis although the physiobiological link is not clearly proven because HIV mortality rate remains high in LMICs, We think that it would be necessary to carry out additional studies to better understand the impact of HIV on epilepsy (if there is one). The pathophysiological mechanisms involved in this association have not yet been well clarified^87^.

#### Meningitis

It is one of the most frequent causes of febrile seizures. The risk of developing epilepsy after an episode of meningitis appears to be very low, but the risk is six times higher among those who start twitching during the acute phase of the disease^88^. In our systematic review, very few studies were identified on this topic. The pathophysiological mechanisms involved in the epileptic process include a series of changes such as an increase in inflammatory cytokines including TNF, in response to a chronic inflammation related to activation of the immune system in response to an endotoxemia^89^.

### Strength and limitations of this study

This is the first study to display such pooled results with worldwide data on comorbidities of epilepsy in LMICs. The application of a transformation method to stabilize logit estimates of proportions reinforces the estimates accuracy. Focusing on studies in the general population increases the external validity of our study.

Nevertheless, this study has some limitations. Firstly, using keywords only in English could be a reason for not identifying more studies conducted in Latin America. The second limitation is the high heterogeneity observed between studies. It is related to differences in methods and sample sizes, as well as the geographic variability of the studied populations. We took it into account by conducting random effects models. We have weighed studies based on a precision estimator, we could have used other methods of weights. We did not examine epilepsy phenotypes, which may underestimate comorbidities in non-convulsive epilepsies. We could have also used other databases such as African Index Medicus, which could have improved our exhaustiveness.

## Conclusion

Through this study we have been able to identify the main comorbidities of epilepsy in LMICs and to determine their relative proportions. Aside from the disease itself, specific factors associated with epilepsy may have an impact on the quality of life of PWE. Most of them are preventable and treatable. The majority of these comorbid conditions share common mechanisms with epilepsy, direct or indirect, causal or through etiologies or common risk factors (genetic or environmental). It is therefore necessary to conduct further studies with valid approaches to understand their impact. Results also highlight the importance of comprehensive care for PWE, ensuring that patients and their caregivers receive correct information about the multifactorial aspect of care for epilepsy is crucial for the quality of care^90–138^.

## Supplementary information

Supplementary tables 1 and 2.
