# Supplementary material for: Comorbidities of epilepsy in low and middle-income countries: systematic review and meta-analysis
Source: Sci Rep. 2020 Jun 2;10:9015. doi: 10.1038/s41598-020-65768-6 (PMC7265529; doi:10.1038/s41598-020-65768-6)
Supplement: Supplementary file 1 — Supplementary tables 1 and 2. [file 41598_2020_65768_MOESM1_ESM.pdf]

Comorbidities of epilepsy in low and middle-income countries: systematic review and meta-analysis

Aline Muhigwa[1,2*] • Pierre-Marie Preux[1] • Daniel Gérard[1] • Benoit Marin[1] • Farid Boumediène[1] • Charles Ntamwira[2]

Chung-Huang Tsai[3]

Supplementary Table 1: Proportion of psychosocial comorbidities in LMICs

Author	Country	year	n PWE	Mar Discr. %	Job Discr. %
**Crepin et al.**[97]	Benin	2007	131	-	37.4
**Prischich et al.**[98]	Cameroon	2004	18	88.9	16.7
**Kariuki et al.**[99]	Tanzania, Kenya, Ghana, South Africa	2014	2170	44.6	29.8
**Kariuki et al.**[95]	Tanzania, Kenya, Ghana, South Africa	2015	-	-	78.0
**Kariuki et al.**[95]	Tanzania, Kenya, Ghana, South Africa	2015	1426	77.0	-
**Rwiza et al.**[99]	Tanzania	1993	203	62.1	-
**Matuja et al.**[100]	Tanzania	2001	174	78.9	-
**Ibinga et al.**[101]	Gabon	2015	83		21.4
**Bernet-Bernady et al.**[102]	CAR	1997	187	79.1	-
**Stefanello et al.**[103]	Brazil	2010	153	49.0	7.4
**Montano et al.**[104]	Peru	2005	29	79.1	21.4

Mar Discr: Mariage discrimination
Job Discr: Job discrimination
PWE: people with epilepsy
CAR:Central African Republic

Supplementary Table 2: Proportion of perinatal events in LMICs

Author	Country	Year	N	n PWE	PT	PI	PNA	DP
**Osuntokun et al.[103]**	Nigeria	1987	18954	101	2.0	-	-	-
**Goudsmit et al[104]**	Liberia	1983	44436	123	3.0	-	-	-
**Debouverie et al.[108]**	Burkina-Faso	1993	16627	-	10.0	-	-	-
**Kariuki et al.[113]**	South Africa	2014	584586	2170	5.6	-	-	-
**Ngugi et al.[2]**	South Africa	2013	2262	971	5.8	-	12.1	14.8
**Burton et al.[96]**	Tanzania	2011	225	112		-	14.3	-
**Rwiza et al.[99]**	Tanzania	1992	18183	207	1.0	-	-	-
**Matuja et al.[100]**	Tanzania	2001	348	174		-	12.1	-
**Haimanot et al.[111]**	Ethiopia	1997	61686	139	1.4	-	-	-
**Wagner et al.[93]**	South Africa	2014	552	292	5.0	-	-	-
**Chen et al.[112]**	Taiwan	2006	13663	37	5.4	-	-	-
**Hackett et al.[108]**	India	1997	365	365	8.4	7.5	-	-
**Sampaio et al.[109]**	Brazil	2009	22013	94	-	-	18.8	-
**Velez et al[114].**	Colombia	2006	8910	92	-	-	8.6	-
**Medina et al.[110]**	Honduras	2005	6473	90	7.8	-	-	-
**Sampaio et al.[109]**	Brazil	2009	22013	94	-	1.9	-	-

PWE: people with epilepsy
PT: perinatal traumas
PI: perinatal infections
PNA: perinatal and neonatal asphyxia
DP: difficult pregnancies
